# Cross-modal signatures in maternal speech and singing

**DOI:** 10.3389/fpsyg.2013.00811

**Published:** 2013-11-01

**Authors:** Sandra E. Trehub, Judy Plantinga, Jelena Brcic, Magda Nowicki

**Affiliations:** Music Development Laboratory, Department of Psychology, University of Toronto MississaugaMississauga, ON, Canada

**Keywords:** speech, singing, infants, adults, cross-modal, identification

## Abstract

We explored the possibility of a unique cross-modal signature in maternal speech and singing that enables adults and infants to link unfamiliar speaking or singing voices with subsequently viewed silent videos of the talkers or singers. In Experiment 1, adults listened to 30-s excerpts of speech followed by successively presented 7-s silent video clips, one from the previously heard speaker (different speech content) and the other from a different speaker. They successfully identified the previously heard speaker. In Experiment 2, adults heard comparable excerpts of singing followed by silent video clips from the previously heard singer (different song) and another singer. They failed to identify the previously heard singer. In Experiment 3, the videos of talkers and singers were blurred to obscure mouth movements. Adults successfully identified the talkers and they also identified the singers from videos of different portions of the song previously heard. In Experiment 4, 6− to 8-month-old infants listened to 30-s excerpts of the same maternal speech or singing followed by exposure to the silent videos on alternating trials. They looked longer at the silent videos of previously heard talkers and singers. The findings confirm the individuality of maternal speech and singing performance as well as adults' and infants' ability to discern the unique cross-modal signatures. The cues that enable cross-modal matching of talker and singer identity remain to be determined.

## Introduction

Mothers around the world talk and sing to their pre-verbal infants (Trehub et al., 1993; Trehub and Trainor, 1998), presumably to gain their attention, modulate their arousal, share feelings, and strengthen dyadic ties (Fernald, 1992; Shenfield et al., 2003; Trehub et al., 2010). Maternal or infant-directed (ID) speech is generally regarded as a distinct speech register or genre (Fernald, 1992; Papoušek, 1994) although some consider it to be little more than highly expressive speech—happier, more loving, and more comforting than typical adult-directed (AD) speech (e.g., Kitamura and Burnham, 1998; Trainor et al., 2000; Singh et al., 2002). Indeed, the characteristically happy manner of North American ID speech shares some features with vocal elation or high-arousal happiness in AD speech (Banse and Scherer, 1996).

Research on ID speech has focused primarily on its acoustic features across languages and cultures (e.g., Ferguson, 1964; Grieser and Kuhl, 1988; Fernald et al., 1989) and secondarily on its consequences for infant attention, affect, and learning (e.g., Fernald, 1985; Werker and McLeod, 1989; Papoušek et al., 1990; Thiessen et al., 2005). The exaggerated pitch contours, rhythmicity, and repetitiveness of ID speech give it a notably musical flavor (Fernald, 1989; Trehub, 2009). In fact, the acoustic features of ID speech are more similar to those of ID song than to AD speech (Corbeil et al., 2013), leading some scholars to characterize ID speech as a form of music (Brandt et al., 2012). Differences in syntactic and semantic aspects of ID and AD speech, although substantial (e.g., Ferguson, 1964; Papoušek, 1994), presumably have less impact on pre-verbal listeners than do expressive aspects of such speech. In fact, there is evidence that the expressivity of ID speech to 12-month-olds is somewhat attenuated as compared with speech to younger infants (Stern et al., 1983; Kitamura and Burnham, 2003).

With attention focused largely on common features and cultural variations of ID speech, there has been relatively little interest in questions of individuality. Bergeson and Trehub (2007) found, however, that mothers used individually distinctive melodies, or *signature tunes*, in their speech to infants. In two recording sessions separated by a week or so, they found that mothers repeatedly used a small set of individually distinctive tunes (i.e., specific interval sequences that were unrelated to musical scales), varying the verbal content that accompanied those tunes. Such tunes—their pitch patterns and rhythms—could provide important cues to speaker identity. Just as communicative intentions are more transparent in ID than in AD speech (Fernald, 1989), even across disparate cultures (Bryant and Barrett, 2007), prosodic cues to identity may be more transparent in ID than in AD speech. It is unclear, however, whether phonetic or articulatory cues (i.e., talkers' idiolect) are individually distinctive in ID speech, as they are in AD speech (Fellowes et al., 1997; Sheffert et al., 2002).

In interactions with infants, mothers also use exaggerated facial (Chong et al., 2003) and body gestures (Brand et al., 2002; Brand and Shallcross, 2008) that feature greater repetitiveness and range of motion than AD gestures. To date, however, there has been no attempt to ascertain whether these visual aspects of ID speech are individually distinctive. Adults recognize familiar individuals from facial motion (Hill and Johnston, 2001), which provides visual correlates of prosody and articulation, and from point-light displays derived from the teeth, tongue, and face of talkers (Rosenblum et al., 2007), which provide visual cues to idiolect. Adults perform modestly but above chance levels in a delayed matching-to-sample task involving unfamiliar voices and silent videos from the same or different utterances (Kamachi et al., 2003; Lachs and Pisoni, 2004; Lander et al., 2007). In one condition, Kamachi et al. (2003) and Lander et al. (2007) presented adults with a scripted utterance followed by successively presented silent videos, one from the previously heard speaker articulating the same utterance (or a different scripted utterance in another condition) and the other from a different speaker. Performance was somewhat better for cross-modal matching of the same utterances than for different utterances. Performance was equivalent, however, for participants who experienced the stimuli in reverse order, for example, a silent video followed by two successively presented utterances. The results imply the presence of signature features in the audible and visible aspects of speech, perhaps based on rhythmic structures or expressiveness (Lander et al., 2007).

In previous research, the importance of temporal cues was indicated by adults' inability to match audible and visible aspects of speech when the stimuli were played backward rather than forward (Kamachi et al., 2003; Lachs and Pisoni, 2004). The manner or style of speech seems to make a critical contribution to performance. For example, changing the manner from statement to question form, from conversational style to clear (i.e., carefully articulated) speech, or from conversational to rushed casual speech significantly reduces identification accuracy (Lander et al., 2007). By contrast, electronic speeding or slowing of speech does not impair the accuracy of cross-modal matching (Lander et al., 2007), which implies that relational rather than absolute timing cues are implicated.

The goal of the present research was to ascertain whether auditory and visible aspects of maternal speech and song have a common signature that is perceptible to adults who are unfamiliar with the talkers and singers. The perceptibility of that signature would enable adults, perhaps even infants, to match auditory and visual components of maternal speech and song in the context of a delayed matching-to-sample task. As is the case for ID speech, research on ID song has focused largely on its acoustic features (e.g., Rock et al., 1999; Nakata and Trehub, 2011) and its consequences for infant attention (Trainor, 1996; Tsang and Conrad, 2010; Corbeil et al., 2013), arousal (Shenfield et al., 2003) and learning (Volkova et al., 2006; Lebedeva and Kuhl, 2010). Although mothers perform the same ID songs at nearly identical pitch level and tempo on different occasions (Bergeson and Trehub, 2002), it is unclear whether their performances of different songs exhibit comparable stability and uniqueness. In any case, pitch level and tempo are not considered reliable cues to the identity of speakers (Kunzel, 1989; Lander et al., 2007).

## Experiment 1

In the present experiment, we sought to ascertain whether adults could link person-specific auditory and visual components of ID speech in a delayed matching-to-sample task. The procedure was modeled on that of Kamachi et al. (2003) who found that adults performed no differently when visual images were matched to previously heard voices or voices were matched to previously seen visual images. For our purposes, adults on each trial were exposed to a 30-s sample of natural ID speech from one of two unfamiliar women followed by two silent videos of speech presented sequentially, one from the previously heard woman, the second from the other woman. Their task was to identify which video corresponded to the previously heard speaker. The stimuli in previous face-voice matching studies featured the same scripted words or utterances for all speakers (e.g., Kamachi et al., 2003; Lachs and Pisoni, 2004; Lander et al., 2007) in contrast to the present experiment, which involved maternal speech extracted from natural interactions with infants. As a result, message content differed from one mother to another and for different parts of the discourse of the same mother. In principle, adults would be capable of lipreading some of the verbal content from silently articulating mothers, which necessitated the use of different speech passages from each mother at exposure and test phases. In other words, the verbal content differed from exposure to test and between the two test stimuli (familiar and unfamiliar women).

### Method

The Office of Research Ethics at the University of Toronto approved all research reported here.

#### Participants

The participants were 44 young adults (24 women, 20 men) who were enrolled in an undergraduate course in introductory psychology. All were healthy and free of hearing loss, according to self-report.

#### Apparatus

Testing took place in a double-walled sound-attenuating booth (Industrial Acoustics) with two Audiological GSI loudspeakers located to the left and right of the seated participant at a 45-degree angle. Stimulus presentation and response recording were controlled by customized software (Real Basic) on a Windows workstation and amplifier (Harmon Kardon 3380) located outside the booth. Visual stimuli were presented on a monitor (Dell LCD, 33.5 × 26.5 cm) directly in front of the participants (at a distance of ~1 m), who entered their responses on a hand-held keypad (Targus) connected to the computer.

#### Stimuli

Audio stimuli consisted of 30-s excerpts from previously recorded QuickTime videos (Sony 360X recorder) of mothers talking to their 11- to 12-month-old infants. Video stimuli, which filled the entire screen, were silent 7-s clips from different portions of the original videos (head and shoulders view of mother). Four pairs of mothers were selected from a larger set to minimize within-pair differences in physical appearance (e.g., race, stature, hair style, clothing).

#### Procedure

**Figure 1:**
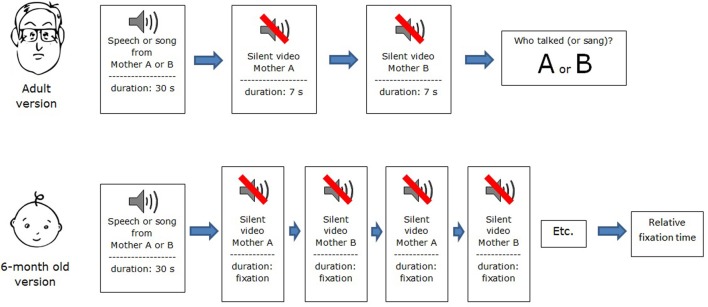
**Flow chart depicting adult and infant versions of the procedure**.

Participants were tested individually in a delayed matching-to-sample task. Before each of the four test trials, they were instructed to listen carefully to the speech excerpt and then to watch the two silent videos in succession. After the second video, static images of the two women from the videos appeared side by side on the monitor, and participants were required to judge which woman had been heard previously. A schematic view of the procedure is presented in Figure 1. Participants entered their choice on a hand-held keypad, which they also used to control the onset of trials. Half of the participants heard the audio excerpts of one woman from each pair and half heard the audio excerpts of the other woman. Matching and non-matching videos were presented in random order.

### Results and discussion

**Figure 2:**
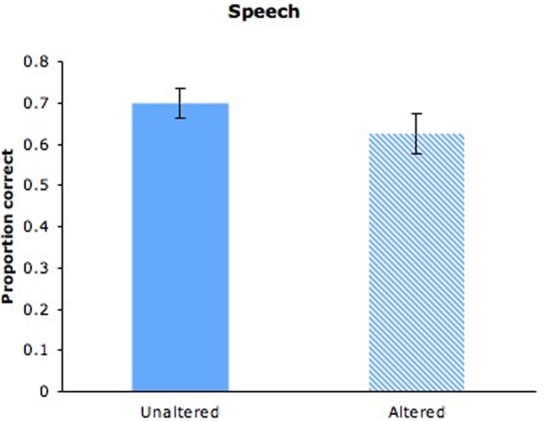
**Adults' proportion of correct responses for maternal speech with unaltered videos (solid bar) or altered videos (hatched bar)**. Error bars are standard errors.

As can be seen in Figure 2 (solid bar), adults matched person-specific auditory and visual aspects of speech imperfectly (*M* = 0.70, *SD* = 0.24) but well above the proportion correct expected by chance (0.50), *t*_(43)_ = 19.292, *p* < 0.001. Moreover, women did not perform better than men, and performance did not differ across stimulus pairs. Adults' success at identifying previously heard maternal speakers on the basis of dynamic visual depictions of those speakers confirms the presence of individually distinctive cross-modal features in maternal speech. The nature of those features remains to be determined. Although two pairs of mothers exhibited differences in speaking rate (*M* = 2.77 vs. 2.03 and 2.90 vs. 1.57 syllables per sec), the other two pairs exhibited little difference (*M* = 2.63 vs. 2.67 and 2:63 vs. 2.60 syllables per sec). Nevertheless, participants performed no better on pairs that differed in speaking rate than those that did not, indicating that speech rate could not account for successful matching in this delayed matching-to-sample task.

The present findings add to the growing literature on adults' perception of face-voice relations in speech (Kamachi et al., 2003; Lachs and Pisoni, 2004; Munhall and Buchan, 2004; Rosenblum et al., 2006; Lander et al., 2007). They are consistent with the view that aspects of speech manner, independent of verbal content and modality, are person-specific. The unique contribution of the present experiment is its focus on ID speech and the use of speech from natural interactions rather than scripted portrayals. Despite the fact that ID speech to pre-verbal infants has many common features within and across cultures (Ferguson, 1964; Grieser and Kuhl, 1988; Fernald et al., 1989), it retains individually distinctive acoustic features that have perceptible visual correlates.

## Experiment 2

Our goal here was to ascertain whether adults could link person-specific auditory and visual components of ID singing in the delayed matching-to-sample task of Experiment 1. It is clear that visual features of sung performances carry music-related information. For example, singers provide cues to the magnitude of isolated intervals (i.e., two successive notes) by their facial and head movements (Thompson et al., 2010). Listeners' judgment of the affective connotation of such intervals is influenced by singers' facial expression (Thompson et al., 2008). To date, however, no study has investigated cross-modal identification of unfamiliar singers. On each trial of the present study, adults were exposed to a 30-s excerpt from an ID song performed by one of two unfamiliar women followed by two silent videos of a different song, presented one after the other. One silent video was from the previously heard singer, the other from the unheard singer. Their task was to identify which video corresponded to the previously heard singer.

### Method

#### Participants

The participants were 20 young adults (14 women, 6 men), mostly undergraduates. All were healthy and free of hearing loss, according to self-report.

#### Apparatus

The apparatus was the same as that in Experiment 1.

#### Stimuli

Singing excerpts from four pairs of mothers were roughly 30 s in duration and were drawn from maternal interactions with infants. The pairs were selected to minimize gross differences in appearance. Silent video excerpts from each mother were from different songs to preclude the use of lipreading cues to song identity. Because mothers sang well-known nursery songs and different mothers sang different songs, song identity and therefore singer identity could have been obvious from visual features alone.

#### Procedure

The procedure was identical to that of Experiment 1 except for the use of maternal singing rather than speech (see Figure 1).

#### Results and discussion

**Figure 3:**
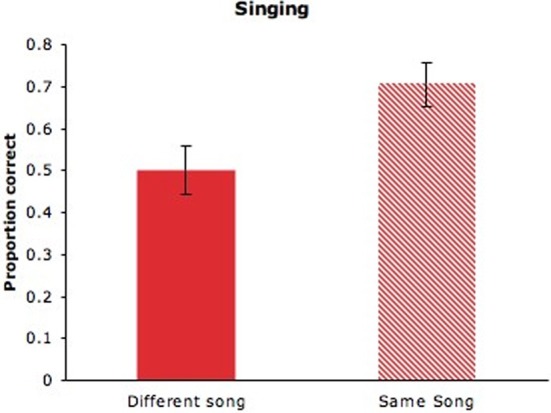
**Adults' proportion of correct responses for maternal singing with unaltered videos from different songs (solid bar) or altered videos from the same song (hatched bar)**. Error bars are standard errors.

Adults' selection of the matching videos (*M* = 0.5, *SD* = 0.60) was at chance levels (see Figure 3, solid bar), indicating that different maternal songs did not provide a common audiovisual signature, as was the case for maternal speech in Experiment 1. Previous research revealed that altering the manner of speech (e.g., statement to question; conversational speech to clear speech) between auditory familiarization and visual test impaired adults' performance on the delayed matching-to-sample task (Lander et al., 2007). When singing to infants, mothers may alter their performing style across songs to highlight the distinctiveness of each song or their own expressive intentions. It is possible, however, that cross-modal correspondences in maternal singing would be evident in the context of specific songs.

## Experiment 3

Adults successfully matched the speech of specific mothers to subsequent silent depictions of different utterances (Experiment 1). Interestingly, they failed to do so with audible and visible (silent) excerpts from different songs. Because the auditory and visual excerpts of speech and singing differed from exposure to test, correct person identification could not be achieved by relating the heard message to the lipread content. Prosody is known to contribute to person identification (Lander et al., 2007), as does the idiosyncratic manner of articulation or idiolect (Fellowes et al., 1997; Lachs and Pisoni, 2004) in auditory, visual, and audiovisual contexts. Prosodic and articulation features were available to participants in Experiments 1 and 2 and to the participants in previous studies of cross-modal identification (Kamachi et al., 2003; Lachs and Pisoni, 2004; Lander et al., 2007). In the present experiment, we asked whether adults could link person-specific auditory and visual components of ID speech and singing with mouth movements occluded. With lipreading cues eliminated, it was possible to examine adults' ability to link auditory and visual features from different portions of the same song rather than different songs (Experiment 2).

### Method

#### Participants

The participants were 28 young adults (20 women, 8 men), mainly undergraduates, who were healthy and free from hearing loss, according to self-report.

#### Apparatus and stimuli

The apparatus was as described in Experiment 1. The audio excerpts of maternal speech were identical to those used in Experiment 1. The video excerpts were also the same except that Adobe Premiere Pro software was used to blur the mouth region of each speaker frame by frame. The audio excerpts of maternal singing were those used in Experiment 2. The video excerpts differed, however, in that they were selected from different portions of the same song. Adobe Premiere Pro software was used in a comparable manner to blur the mouth region of each singer.

#### Procedure

Participants were tested individually and in the same manner as in Experiments 1 and 2. Speech and singing trials were presented in blocks, and trials within blocks were randomized for each participant. On each trial, matching and non-matching video excerpts (i.e., same or different person) were presented in random order. The first trial block (speech or singing) and the first stimulus within blocks were counterbalanced across participants. Each participant completed eight test trials (i.e., audio excerpts from four different speakers and four different singers).

#### Results and discussion

**Figure 4:**
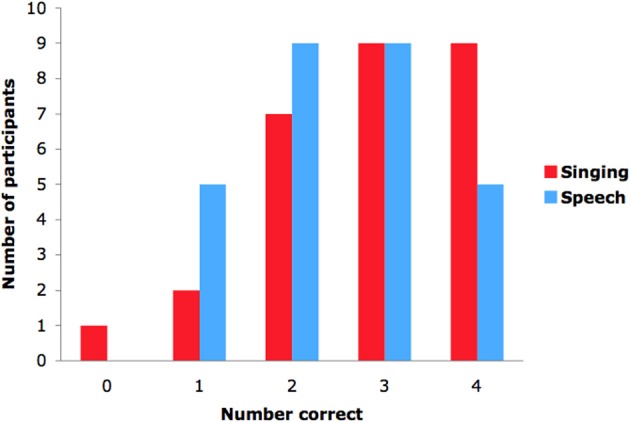
**Number of adults who obtained scores of 0–4 correct on the speech and singing tasks in Experiment 3**.

As can be seen in Figures 2 and 3 (hatched bars), adults succeeded in matching the altered video to audio samples of speech (*M* = 0.63, *SD* = 0.25), *t*_(27)_ = 2.646, *p* = 0.013, and singing (*M* = 0.71, *SD* = 0.27), *t*_(27)_ = 3.986, *p* < 0.001, and performance did not differ across speech and singing, *F*_(1, 26)_ = 0.090, n.s. In other words, adults successfully identified the previously heard speaker and singer on the basis of dynamic visual cues. The absence of cues from the mouth region did not significantly impair adults' ability to identify the speaker, as revealed by comparisons between the present speech condition and that of Experiment 1, *F*_(1, 69)_ = 1.344, n.s. It is likely, then, that prosodic cues and visual correlates of those cues were largely responsible for adults' success on this task. As can be seen in Figure 4, which displays the number of individuals who obtained scores of 0–4 on speaking and singing tasks, there was considerable variation in performance. One might expect individuals who perform well on speaker identification to perform well on singer identification, but performance on speaking and singing blocks was uncorrelated, *r*_(26)_ = −0.017, *p* = 0.932.

Recall that adults in Experiment 2 failed to identify the singers from video portions of different songs. Adults' performance in the present experiment on auditory and visual excerpts from the same song significantly exceeded their performance in Experiment 2 involving visual excerpts from different songs, *F*_(1, 46)_ = 6.949, *p* < 0.01. Unlike professional singers, mothers and other occasional singers may not have a uniform singing style, resulting in potential variations in style or manner across songs. For mothers, in particular, song performances may have different expressive intentions, for example, attention capture in some instances (e.g., *If You're Happy and You Know It*) and attention maintenance (e.g., *Twinkle, Twinkle*) or soothing (e.g., lullabies) in others. In any case, adults' ability to match audible to visible features from different portions of the same song confirms the presence of cross-modal cues to identity.

Lander et al. (2007) speculate that global aspects of expressiveness rather than single acoustic features underlie cross-modal matching in speech, but they did not attempt to quantify gradations in expressiveness. In a supplementary experiment, we had 15 undergraduates rate individual audio and silent video (unblurred) excerpts from each mother on a scale from 1 or neutral to 5 or very expressive/animated. Mean ratings of expressiveness for the four pairs of talking mothers and the four pairs of singing mothers (same song) are shown in Table 1. Although variations in rated expressiveness were evident across mothers, higher ratings of vocal expressiveness were not reliably associated with higher ratings of visual expressiveness. In other words, a mother who spoke or sang more expressively than her paired counterpart did not appear to be more visually expressive than the other mother.

**Table 1:** **Adults' mean expressiveness ratings (and standard deviations) of audio and video excerpts from each mother on a 5-point scale (1 = neutral, 5 = highly animated)**.

**Talking pairs**	**Audio Mom A**	**Video Mom A**	**Audio Mom B**	**Video Mom B**
1	4.8 (0.56)	3.33 (1.23)	2.47 (1.13)	1.93 (0.70)
2	2.68 (0.98)	1.07 (0.26)	1.87 (0.83)	3.23 (0.90)
3	3.93 (1.16)	1.43 (0.50)	2.27 (0.88)	1.77 (0.62)
4	3.40 (1.06)	1.20 (0.41)	4.00 (1.00)	3.50 (1.05)
**Singing pairs**	**Audio Mom A**	**Video Mom A**	**Audio Mom B**	**Video Mom B**
1	3.93 (0.80)	4.17 (0.79)	3.93 (0.96)	3.00 (1.25)
2	2.80 (0.86)	3.27 (0.96)	3.47 (0.64)	3.20 (0.78)
3	4.33 (0.90)	3.63 (0.81)	2.67 (0.72)	2.93 (0.70)
4	3.73 (0.96)	3.67 (0.98)	2.87 (0.83)	3.07 (0.80)

Ratings of speech are presented in the upper section and ratings of singing in the lower section. Columns indicate ratings for different pairs of mothers (1–4) and rows indicate ratings for each pair (Mom A, Mom B). Ratings of talking are for the unaltered excerpts, as in Experiments 1 and 4. Ratings of singing are for unaltered excerpts of the same song, as in Experiment 4.

## Experiment 4

The findings of Experiments 1 and 3 confirmed the presence of unspecified cues to identity in auditory and visual aspects of maternal speech and singing. Recall that discernible cues to identity were found only within but not across songs. In the present experiment we investigated infants' ability to make use of cross-modal cues to identity.

In the early postnatal period, infants differentiate their mother's face from that of a stranger on the basis of static or dynamic images (Sai and Bushnell, 1988). They also differentiate the mother's voice from that of a stranger (DeCasper and Fifer, 1980). At 8 but not 4 months of age, they match auditory and visual cues to gender (Patterson and Werker, 2002), presumably on the basis of acquired knowledge of intermodal correspondences. They integrate emotional information from the face and voice, as indicated by ERP responses to simultaneously presented faces and voices (happy or angry) that are emotionally incongruent (Grossmann et al., 2006). The aforementioned unimodal and intermodal discriminations depend on learning. Nevertheless, infants perceive some cross-modal correspondences that may be independent of learning, arising from as yet unspecified amodal cues. For example, 4- to 5-month-old infants look longer at one of two simultaneously presented visual articulatory displays that matches a repeating vowel sound (/a/ or /i/) presented simultaneously and synchronously (Kuhl and Meltzoff, 1982; Patterson and Werker, 1999, 2002). Infants seem to perceive some connection between mouth shape and vowel category, perhaps because of redundant amodal cues (Bahrick et al., 2004). Remarkably, 6-month-old infants also perceive the links between syllables that they hear (/ba/ or /va/) and dynamic visual images presented before and after the auditory stimuli (Pons et al., 2009). By 10–12 months of age, they link the sounds of their native language to dynamic images of that language, indicating their perception of amodal cues to the identity of a familiar language (Lewkowicz and Pons, 2013).

The focus of the present experiment was on audiovisual cues to identity, as in Experiments 1–3. In contrast to previous cross-modal matching tasks with infants, which usually featured simultaneous visual displays (Kuhl and Meltzoff, 1982; Patterson and Werker, 1999, 2002; Pons et al., 2009; Lewkowicz and Pons, 2013), we used sequential presentation of visual stimuli. The procedure was in line with Experiments 1 and 2, with adjustments to accommodate the needs of 6- to 8-month-old participants. On the basis of individual identification across species (Ghazanfar et al., 2007; Pollard and Blumstein, 2011), one might expect some cues to identity—auditory, visual, and audiovisual—to be extracted automatically and effortlessly, even in early life.

Infants were tested with the familiarization-preference procedure (e.g., Hannon and Trehub, 2005; Plantinga and Trehub, 2013), which was modified to accommodate cross-modal matching. The procedure was similar, in some respects, to the intermodal matching procedure used by Pons et al. (2009), such as auditory stimuli presented separately from visual stimuli, but it differed in several respects including the sequential presentation of visual stimuli. First, infants were exposed to 30-s samples of ID speech or singing after which they received silent videos of the previously heard speaker or singer and another speaker or singer on alternating trials (see Figure 1). In other words, they saw the silent video of the previously heard speaker on every other trial and the silent video of the unheard speaker on intervening trials. If infants perceived amodal cues to identity in the auditory and visual excerpts, they should exhibit differential attention to the video excerpts. For example, they could look longer at videos of the familiar or previously heard speaker or singer or at the videos of the unheard speaker or singer. Infants' success, if evident, would stem from implicit memory for amodal cues, in contrast to adults, who might have explicit memory for person-specific features. Rhythmic factors could be implicated in both cases.

### Method

#### Participants

The participants consisted of a total of 144 infants 6–8 months of age, 48 (*M* = 30.08 weeks, *SD* = 3.16; 25 girls, 25 boys) tested on audio and visual samples of speech, 48 (*M* = 31.41 weeks, *SD* = 3.54; 23 girls, 25 boys) on singing samples with videos from different songs, and 48 (*M* = 32.73, *SD* = 1.80; 21 girls, 27 boys) on the same singing samples with videos from different portions of the same song. All infants were healthy, born at term, and had no personal history of ear infections or family history of hearing loss, according to parental report.

#### Apparatus and stimuli

Infants were tested in a dimly lit sound-attenuating booth with the equipment described in Experiment 1 except for the presence of two additional monitors and a camcorder (Sony 360X) that transmitted images of the infant to the experimenter outside the booth. Infants were seated on their mother's lap facing the central monitor, with two other monitors 1 m away and at a 45-degree angle to their left and right. Parents wore headphones with masking music to prevent them from hearing the auditory stimuli presented to infants. Because of limited numbers of 6-month-old infants available at the time of testing, only three of the four pairs of stimuli from Experiments 1 and 2 (selected for best audio and video quality) were used. The video stimuli for the speech and singing segments were roughly 30 s in duration and were unaltered (i.e., no blurring of mouth area, as in Experiment 3). An experimenter outside the booth viewed the infant on a monitor and maintained a continuous record of infant looking to and away from the side monitors.

#### Procedure

Infants were first familiarized with the audio segments of speech or singing stimuli for 30 s during which time a silent video of a rotating globe was presented to help maintain infants' attention. Infants had 15 s of familiarization with the auditory stimulus paired with the silent video on one side followed by 15 s of the same auditory and visual stimuli on other side. Immediately after the familiarization phase, infants' attention was attracted to one of the side monitors by a flashing light on that monitor. When infants looked at that monitor, a silent video of the relevant condition (speech, different song, same song) was presented and continued to play until they looked away for 2 s. Infants' attention was then attracted to the monitor on the other side, and the contrasting silent speech or singing video from the same condition was presented until infants looked away for 2 s. The two silent video trials continued in alternation for a total of 10 trials. Half of the infants tested on each pair of speech or singing stimuli were familiarized with the audio sample of one mother and half with the audio sample of the other mother. In addition, the order of videos (target mother, other mother) and the side of first video trial (left or right) were counterbalanced.

#### Results and discussion

**Figure 5:**
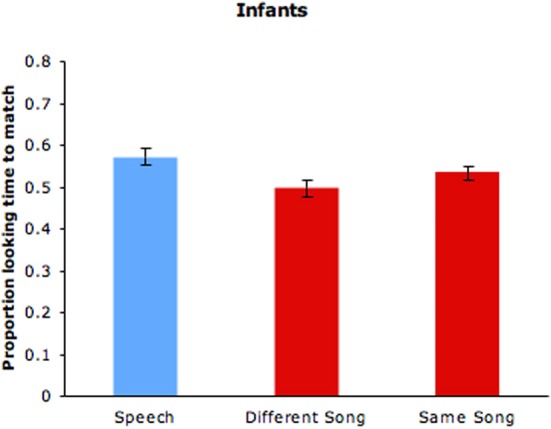
**Infants' proportion of looking time to the speech and singing videos of previously heard speakers and singers (same and different songs)**. Error bars are standard errors.

Because a number of infants in the speech condition failed to complete the full 10 trials, 6 trials (3 with each of the two video stimuli) were used for all infants in that condition. As can be seen in Table 1, the silent talking videos were rated lower in expressiveness than the silent singing videos. The full 10 trials were used in the singing conditions and are reported here. Proportions of infant looking time to the matching silent videos of speakers and singers in the three conditions are shown in Figure 5. Proportion of looking at the videos of previously heard speakers (*M* = 0.573, *SD* = 0.128) significantly exceeded chance levels (0.5), *t*_(47)_ = 3.64, *p* = 0.001, confirming infants' detection of cross-modal cues to speaker identity. By contrast, proportion of looking to the matching silent videos from different songs (*M* = 0.497) was at chance (see Figure 2). For videos featuring different portions of the same song, however, proportion of looking at the matching videos of previously heard singers (*M* = 0.534, *SD* = 0.116) significantly exceeded chance levels, *t*_(47)_ = 2.032, *p* = 0.048. Differences in infant looking times are modest, but they are comparable to the levels reported in other familiarization-preference studies with 6-month-old infants that involve sequential presentation of stimuli (e.g., Hannon and Trehub, 2005). Overall, the findings from infants paralleled those from adults, with infants detecting cross-modal cues to identity for ID speech and for different portions of the same ID song but not for different ID songs.

## General discussion

Adults and infants detected cross-modal cues to identity in maternal speech and singing. Adults' success in the present study confirms and extends the available evidence on cross-modal matching of talkers. It indicates that adults can identify maternal talkers from audio and video excerpts presented sequentially even when the excerpts are based on different verbal content (Kamachi et al., 2003; Lander et al., 2007). Previous research indicated that the manner of speech plays an important role such that changing manner across modalities (e.g., statement to question, conversational to clear speech) impairs cross-modal matching of speakers (Lander et al., 2007).

The manner of speech in the present study differed from that of earlier studies not only in its ID status but also in its derivation from natural interactions rather than portrayals. When “conversational” speech was used in previous studies of cross-modal matching (Lander et al., 2007), the adult “actors”' were instructed to memorize and produce a single scripted utterance (“I'm going to the library in the city.”) and to “speak it in their usual natural manner (conversational statement)” (p. 906). By contrast, natural, conversational samples of ID speech in the present study were derived from playful maternal interactions with infants. As a result, the dynamic visual stimuli in each pair were based on speech samples that differed from each other as well as from the auditory stimuli. The range of possible variation across content, style, and modality was considerable. It would be of interest to ascertain whether adults would be capable of matching cross-modal cues to identity when auditory and visual cues are selected from contrasting registers such as conversational ID and AD speech, which vary considerably in expressiveness (Corbeil et al., 2013). Although female college students performed no better than their male counterparts on matching maternal voices to visual gestures, it is possible that mothers would perform better than non-mothers.

In the case of singing, adults perceived cross-modal cues to identity when the auditory and visual excerpts from each singer were from different portions of the same song with mouth movement obscured (Experiment 3) but not from different songs with intact movement (Experiment 2). Because all mothers sang different songs (i.e., songs that they typically sang to their infants), it is possible that adults in the present study simply identified the excerpts belonging to the same song rather than the same singer. Unfortunately, the design of the present study makes it impossible to rule out that interpretation. Identifying a well-known song from one of two silent videos, even with mouth movements obscured, may seem easy, but performance on the cross-modal singing task was modest and not significantly better than that on the speech task. Tempo appears to be an obvious cross-modal cue, but artificially speeding up or slowing down speech between familiarization and test stimuli does not interfere with adults' cross-modal matching (Lander et al., 2007). However, tempo is probably more salient in singing than in speech. In future research, artificial slowing or speeding of the tempo of maternal singing could indicate the relative contribution of absolute (i.e., tempo) and relative duration cues (i.e., rhythm).

Adults succeeded in identifying unfamiliar talkers and singers from cross-modal cues, but their performance in the present study and in earlier studies of talker identification was modest, roughly 70% correct or less. This kind of task is obviously difficult, even with 30-s passages of speech rather than the single words (Lachs and Pisoni, 2004) or single sentences (Kamachi et al., 2003; Lander et al., 2007) used in previous studies. Lachs and Pisoni (2004) argue that cross-modal matching is facilitated by the kinematics of articulation, but that may apply primarily to situations involving common lexical content across modalities. Removal of mouth cues in Experiment 3 did not significantly reduce performance accuracy, which suggests that global prosodic timing or rhythm was the primary amodal cue. Identifying the subtle visual rhythms that accompany speech and singing is an important challenge for the future.

Infants are presumed to use amodal cues when matching repeated vowels (/a/ or /i) to dynamic visual displays presented simultaneously and synchronously (Kuhl and Meltzoff, 1982; Patterson and Werker, 1999, 2002) and when matching repeating consonant-vowel syllables (/ba/ or /va/) to dynamic visual displays presented sequentially (Pons et al., 2009). Infants' use of amodal cues to identity in the present study, which involved sequential presentation of highly complex auditory and visual stimuli, is especially impressive. What did infants retain from the auditory familiarization phase, and what drove their longer looking times to videos of the previously heard speaker or singer? Perhaps adults formed intuitive impressions of the talkers and singers as they listened to the stimuli, even imagining what they might look like. Then they had an opportunity to watch both silent videos before deciding who was more likely to be the previously heard speaker or singer. Our supplementary rating experiment ruled out the most obvious factor in this regard, which was expressiveness or liveliness.

Adults typically have difficulty linking voices to static facial images (Kamachi et al., 2003; Lachs and Pisoni, 2004), but a recent study revealed poor but above-chance performance with static images presented sequentially (Mavica and Barenholtz, 2013). It is possible that adults generate expectations of a speaker's or singer's physical appearance or visual gestures while listening to that person, but infants are unlikely to do so. Nevertheless, the ID talking or singing in the present study primed infants for subsequent engagement with the talker's or singer's dynamic visual images. Something about each woman's ID speech or singing was engaging to infants as well as individually distinctive, memorable, and recognizable across modalities. As noted, global temporal features involving rhythmic prosody (Kamachi et al., 2003; Lander et al., 2007) are more likely candidates than local temporal features involving the fine-grained dynamics of articulation (Patterson and Werker, 1999; Lachs and Pisoni, 2004).

There was no indication that mouth movements contributed to adults' performance (Experiment 3), but they could have affected infants' performance. When exposed to audiovisual speech, 4-month-old infants fixate more on the eyes than on the mouth, 6-month-olds distribute their fixations equally across eye and mouth regions, and 8-month-olds focus more on the mouth than on the eyes (Lewkowicz and Hansen-Tift, 2012). Although there is no evidence that infants extract or retain person-specific cues to articulation, as older children do (Vongpaisal et al., 2010; van Heugten et al., in press), they may capitalize on other idiosyncratic features involving lip movements.

In sum, the present study revealed that mothers provide signature bimodal performances of speech and singing for their pre-verbal infants. Moreover, adults discern cross-modal cues to the identity of maternal speakers and singers and, remarkably, infants do so as well. An important task for future research is to specify the critical bimodal cues for infants and adults.

### Conflict of interest statement

The authors declare that the research was conducted in the absence of any commercial or financial relationships that could be construed as a potential conflict of interest.
